# Supplementary material for: Intellectual conflicts of interest among cardiology and pulmonology clinical practice guidelines
Source: PLoS One. 2023 Jul 10;18(7):e0288349. doi: 10.1371/journal.pone.0288349 (PMC10332620; doi:10.1371/journal.pone.0288349)
Supplement: S1 Table — (PDF) [file pone.0288349.s001.pdf]

S1 Table. Guidelines, Professional Societies, and Intellectual Conflicts of Interests

Guideline Title*	Professional Society	No. Authors	No. Chairs*	No. (%) Authors with any intellectual COI	No. (%) Chairs with any intellectual COI
Clinically Diagnosing Pertussis-associated Cough in Adults and Children[1]	American College of Chest Physicians	5	1	3 (33%)	0 (0%%)
Adult Outpatients With Acute Cough Due to Suspected Pneumonia or Influenza[2]	American College of Chest Physicians	6	0	3 (50%)	0 (*n/a*)
Antithrombotic Therapy for Atrial Fibrillation[3]	American College of Chest Physicians	12	1	9 (75%)	1 (100%)
Classification of Cough as a Symptom in Adults and Management Algorithms[4]	American College of Chest Physicians	4	0	4 (100%)	0 (*n/a*)
Cough Due to TB and Other Chronic Infections[5]	American College of Chest Physicians	5	1	1 (20%)	1 (100%)
Screening for Lung Cancer[6]	American College of Chest Physicians	8	1	6 (75%)	1 (100%)
Therapy for pulmonary arterial hypertension in adults[7]	American College of Chest Physicians	13	3	6 (46%)	2 (67%)
Treatment of Interstitial Lung Disease Associated Cough[8]	American College of Chest Physicians	6	1	2 (33%)	0 (0%)
Diagnosis of Idiopathic Pulmonary Fibrosis[9]	American Thoracic Society	34	5	19 (56%)	4 (80%)
Diagnosis and Treatment of Adults with Community-acquired Pneumonia[10]	American Thoracic Society	15	1	10 (67%)	1 (100%)
Management of Malignant Pleural Effusions[11]	American Thoracic Society	14	3	8 (57%)	1 (33%)
Diagnosis of Primary Ciliary Dyskinesia[12]	American Thoracic Society	30	4	15 (50%)	3 (75%)
Evaluation and Management of Obesity Hypoventilation Syndrome[13]	American Thoracic Society	21	2	9 (43%)	2 (100%)
Home Oxygen Therapy for Children[14]	American Thoracic Society	22	2	11 (50%)	2 (100%)
Microbiological Laboratory Testing in the Diagnosis of Fungal Infections in Pulmonary and Critical Care Practice[15]	American Thoracic Society	11	1	3 (27%)	1 (100%)
The Role of Weight Management in the Treatment of Adult Obstructive Sleep Apnea[16]	American Thoracic Society	16	2	10 (63%)	2 (100%)
Treatment of Drug-Resistant Tuberculosis[17]	American Thoracic Society	35	3	24 (69%)	3 (100%)
Use of Cystic Fibrosis Transmembrane Conductance Regulator Modulator Therapy in Patients with Cystic Fibrosis[18]	Cystic Fibrosis Foundation	14	2	3 (21%)	2 (100%)
British Thoracic Society Guideline for bronchiectasis in adults[19]	British Thoracic Society	22	3	13 (59%)	3 (100%)
British Thoracic Society Guideline for the initial outpatient management of pulmonary embolism[20]	British Thoracic Society	17	1	3 (18%)	0 (0%)
British Thoracic Society Guideline for the investigation and management of malignant pleural mesothelioma[21]	British Thoracic Society	17	2	12 (71%)	2 (100%)
British guideline on the management of asthma[22]	British Thoracic Society	33	2	13 (39%)	2 (100%)
Clinical Practice Guidelines for the Prevention and Management of Pain, Agitation/Sedation, Delirium, Immobility, and Sleep Disruption in Adult Patients in the ICU[23]	Society of Critical Care Medicine	41	2	31 (76%)	2 (100%)
Fluid therapy in neurointensive care patients[24]	European Society of Intensive Care Medicine	22	4	12 (55%)	2 (50%)
European Respiratory Society guidelines on long-term home non-invasive ventilation for management of COPD[25]	European Respiratory Society	20	3	14 (70%)	3 (100%)

S1 Table (cont.)

Guideline Title*	Professional Society	No. Authors	No. Chairs*	No. (%) Authors with iCOI	No. (%) Chairs with any iCOI
Guideline on the Primary Prevention of Cardiovascular Disease[26]	American College of Cardiology	18	2	12 (66%)	2 (100%)
Guideline on the Evaluation and Management of Patients With Bradycardia and Cardiac Conduction Delay[27]	American College of Cardiology	19	1	13 (68%)	1 (100%)
Guideline for the Management of Adults With Congenital Heart Disease[28]	American Heart Association	15	2	13 (87%)	2 (100%)
Guideline on the Management of Blood Cholesterol: A Report of the American College of Cardiology/American Heart Association Task Force on Clinical Practice Guidelines[29]	American Heart Association	24	1	17 (71%)	1 (100%)
Guidelines for the Early Management of Patients With Acute Ischemic Stroke[30]	American Heart Association	19	0	13 (68%)	0 (*n/a*)
Guidelines for the Diagnosis and Management of Acute Pulmonary Embolism[31]	European Society of Cardiology	22	2	19 (86%)	2 (100%)
Guidelines for the diagnosis and management of syncope[32]	European Society of Cardiology	17	1	15 (88%)	1 (100%)
Guidelines for the Management of Cardiovascular Diseases During Pregnancy[33]	European Society of Cardiology	20	2	13 (65%)	2 (100%)
Guidelines on Myocardial Revascularization[34]	European Society of Cardiology	22	2	18 (82%)	2 (100%)
Guidelines for the Management of Arterial Hypertension[35]	European Society of Cardiology	28	2	24 (86%)	2 (100%)
Guidelines for the Diagnosis and Management of Chronic Coronary Syndromes[36]	European Society of Cardiology	25	2	22 (88%)	2 (100%)
Guidelines for the Management of Patients With Supraventricular Tachycardia[37]	European Society of Cardiology	21	3	10 (48%)	2 (100%)
Guidelines on Diabetes, Pre-Diabetes, and Cardiovascular Diseases[38]	European Society of Cardiology	24	2	22 (92%)	2 (100%)
Guidelines for the Management of Dyslipidemias: Lipid Modification to Reduce Cardiovascular Risk[39]	European Society of Cardiology	21	2	19 (90%)	2 (100%)

*All titles are abbreviated. Full list of guidelines with full citation available below

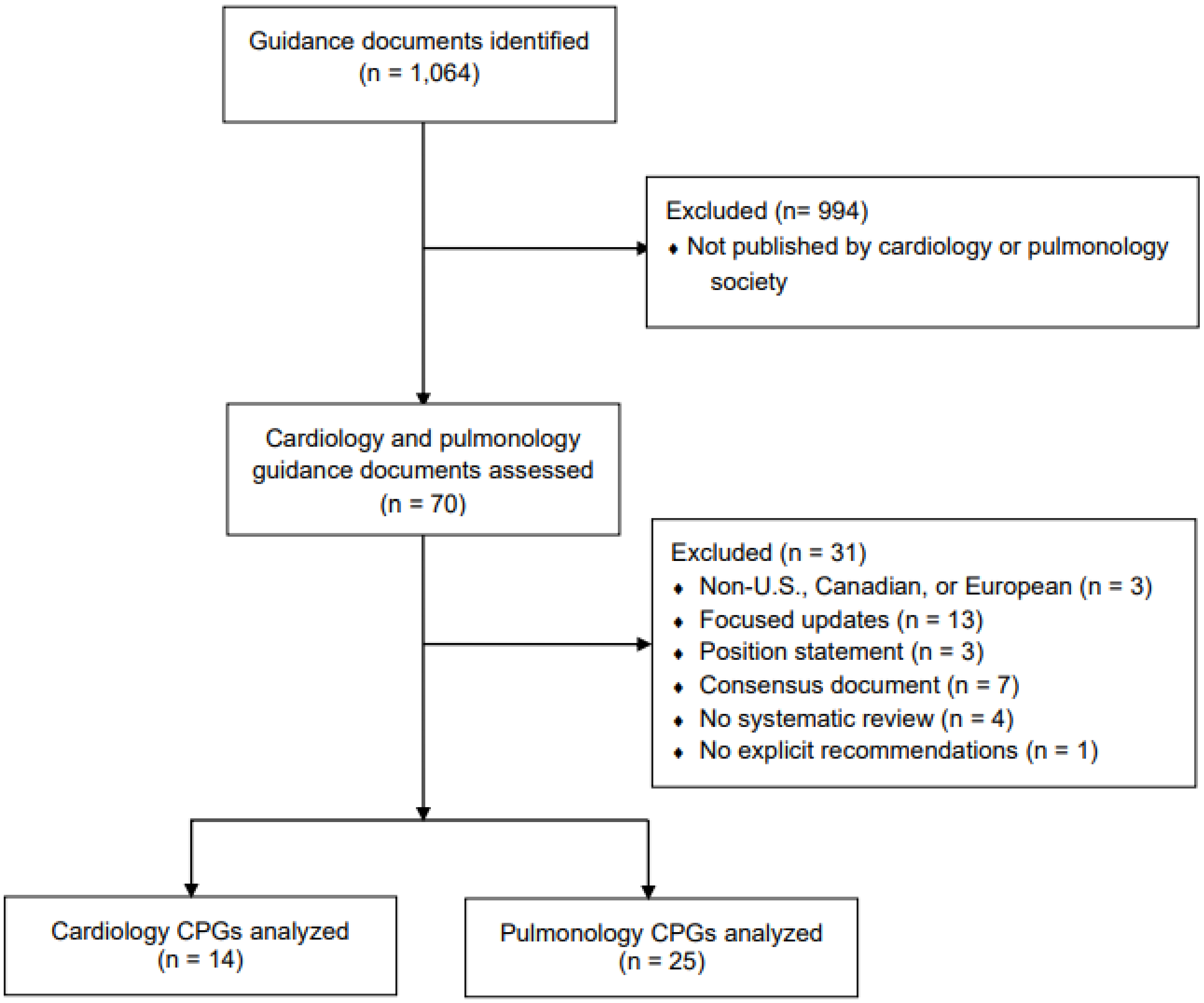

S1 Figure. Identification and assessment of clinical practice guidelines (CPGs)

References – Guideline List

1. Moore A, Harnden A, Grant CC, et al. Clinically Diagnosing Pertussis-associated Cough in Adults and Children: CHEST Guideline and Expert Panel Report. *CHEST*. 2019;155(1):147-154. doi:10.1016/j.chest.2018.09.027

2. Hill AT, Gold PM, Solh AAE, et al. Adult Outpatients With Acute Cough Due to Suspected Pneumonia or Influenza: CHEST Guideline and Expert Panel Report. *CHEST*. 2019;155(1):155-167. doi:10.1016/j.chest.2018.09.016

3. Lip GYH, Banerjee A, Boriani G, et al. Antithrombotic Therapy for Atrial Fibrillation: CHEST Guideline and Expert Panel Report. *CHEST*. 2018;154(5):1121-1201. doi:10.1016/j.chest.2018.07.040

4. Irwin RS, French CL, Chang AB, et al. Classification of Cough as a Symptom in Adults and Management Algorithms: CHEST Guideline and Expert Panel Report. *CHEST*. 2018;153(1):196-209. doi:10.1016/j.chest.2017.10.016

5. Field SK, Escalante P, Fisher DA, et al. Cough Due to TB and Other Chronic Infections: CHEST Guideline and Expert Panel Report. *CHEST*. 2018;153(2):467-497. doi:10.1016/j.chest.2017.11.018

6. Mazzone PJ, Silvestri GA, Patel S, et al. Screening for Lung Cancer: CHEST Guideline and Expert Panel Report. *CHEST*. 2018;153(4):954-985. doi:10.1016/j.chest.2018.01.016

7. Klinger JR, Elliott CG, Levine DJ, et al. Therapy for Pulmonary Arterial Hypertension in Adults: Update of the CHEST Guideline and Expert Panel Report. *CHEST*. 2019;155(3):565-586. doi:10.1016/j.chest.2018.11.030

8. Birring SS, Kavanagh JE, Irwin RS, et al. Treatment of Interstitial Lung Disease Associated Cough: CHEST Guideline and Expert Panel Report. *CHEST*. 2018;154(4):904-917. doi:10.1016/j.chest.2018.06.038

9. Raghu G, Remy-Jardin M, Myers JL, et al. Diagnosis of Idiopathic Pulmonary Fibrosis. An Official ATS/ERS/JRS/ALAT Clinical Practice Guideline. *Am J Respir Crit Care Med*. 2018;198(5):e44-e68. doi:10.1164/rccm.201807-1255ST

10. Metlay JP, Waterer GW, Long AC, et al. Diagnosis and Treatment of Adults with Community-acquired Pneumonia. An Official Clinical Practice Guideline of the American Thoracic Society and Infectious Diseases Society of America. *Am J Respir Crit Care Med*. 2019;200(7):e45-e67. doi:10.1164/rccm.201908-1581ST

11. Feller-Kopman DJ, Reddy CB, DeCamp MM, et al. Management of Malignant Pleural Effusions. An Official ATS/STS/STR Clinical Practice Guideline. *Am J Respir Crit Care Med*. 2018;198(7):839-849. doi:10.1164/rccm.201807-1415ST

12. Shapiro AJ, Davis SD, Polineni D, et al. Diagnosis of Primary Ciliary Dyskinesia. An Official American Thoracic Society Clinical Practice Guideline. *Am J Respir Crit Care Med*. 2018;197(12):e24-e39. doi:10.1164/rccm.201805-0819ST

13. Mokhlesi B, Masa JF, Brozek JL, et al. Evaluation and Management of Obesity Hypoventilation Syndrome. An Official American Thoracic Society Clinical Practice Guideline. *Am J Respir Crit Care Med*. 2019;200(3):e6-e24. doi:10.1164/rccm.201905-1071ST

14. Hayes D, Wilson KC, Krivchenia K, et al. Home Oxygen Therapy for Children. An Official American Thoracic Society Clinical Practice Guideline. *Am J Respir Crit Care Med*. 2019;199(3):e5-e23. doi:10.1164/rccm.201812-2276ST

15. Hage CA, Carmona EM, Epelbaum O, et al. Microbiological Laboratory Testing in the Diagnosis of Fungal Infections in Pulmonary and Critical Care Practice. An Official American Thoracic Society Clinical Practice Guideline. *Am J Respir Crit Care Med*. 2019;200(5):535-550. doi:10.1164/rccm.201906-1185ST

16. Hudgel DW, Patel SR, Ahasic AM, et al. The Role of Weight Management in the Treatment of Adult Obstructive Sleep Apnea. An Official American Thoracic Society Clinical Practice Guideline. *Am J Respir Crit Care Med*. 2018;198(6):e70-e87. doi:10.1164/rccm.201807-1326ST

17. Nahid P, Mase SR, Migliori GB, et al. Treatment of Drug-Resistant Tuberculosis. An Official ATS/CDC/ERS/IDSA Clinical Practice Guideline. *Am J Respir Crit Care Med*. 2019;200(10):e93-e142. doi:10.1164/rccm.201909-1874ST

18. Ren CL, Morgan RL, Oermann C, et al. Cystic Fibrosis Foundation Pulmonary Guidelines. Use of Cystic Fibrosis Transmembrane Conductance Regulator Modulator Therapy in Patients with Cystic Fibrosis. *Ann Am Thorac Soc*. 2018;15(3):271-280. doi:10.1513/AnnalsATS.201707-539OT

19. Hill AT, Sullivan AL, Chalmers JD, et al. British Thoracic Society Guideline for bronchiectasis in adults. *Thorax*. 2019;74(Suppl 1):1-69. doi:10.1136/thoraxjnl-2018-212463

20. Howard LSGE, Barden S, Condliffe R, et al. British Thoracic Society Guideline for the initial outpatient management of pulmonary embolism (PE). *Thorax*. 2018;73(Suppl 2):ii1-ii29. doi:10.1136/thoraxjnl-2018-211539

21. Woolhouse I, Bishop L, Darlison L, et al. British Thoracic Society Guideline for the investigation and management of malignant pleural mesothelioma. *Thorax*. 2018;73(Suppl 1):i1-i30. doi:10.1136/thoraxjnl-2017-211321

22. Scottish Intercollegiate Guideliens Network, British Thoracic Society. British guideline on the management of asthma. SIGN 158. Published July 2019. https://www.sign.ac.uk/media/1773/sign158-updated.pdf

23. Devlin JW, Skrobik Y, Gélinas C, et al. Clinical Practice Guidelines for the Prevention and Management of Pain, Agitation/Sedation, Delirium, Immobility, and Sleep Disruption in Adult Patients in the ICU. *Crit Care Med*. 2018;46(9):e825. doi:10.1097/CCM.0000000000003299

24. Oddo M, Poole D, Helbok R, et al. Fluid therapy in neurointensive care patients: ESICM consensus and clinical practice recommendations. *Intensive Care Med*. 2018;44(4):449-463. doi:10.1007/s00134-018-5086-z

25. Ergan B, Oczkowski S, Rochwerg B, et al. European Respiratory Society guidelines on long-term home non-invasive ventilation for management of COPD. *Eur Respir J*. 2019;54(3):1901003. doi:10.1183/13993003.01003-2019

26. Arnett DK, Blumenthal RS, Albert MA, et al. 2019 ACC/AHA Guideline on the Primary Prevention of Cardiovascular Disease: A Report of the American College of Cardiology/American Heart Association Task Force on Clinical Practice Guidelines. *Circulation*. 2019;140(11). doi:10.1161/CIR.0000000000000678

27. Kusumoto FM, Schoenfeld MH, Barrett C, et al. 2018 ACC/AHA/HRS guideline on the evaluation and management of patients with bradycardia and cardiac conduction delay: A Report of the American College of Cardiology/American Heart Association Task Force on Clinical Practice Guidelines and the Heart Rhythm Society. *Heart Rhythm*. 2019;16(9):e128-e226. doi:10.1016/j.hrthm.2018.10.037

28. Stout KK, Daniels CJ, Aboulhosn JA, et al. 2018 AHA/ACC Guideline for the Management of Adults With Congenital Heart Disease: A Report of the American College of Cardiology/American Heart Association Task Force on Clinical Practice Guidelines. *Circulation*. 2019;139(14):e698-e800. doi:10.1161/CIR.0000000000000603

29. Grundy SM, Stone NJ, Bailey AL, et al. 2018 AHA/ACC/AACVPR/AAPA/ABC/ACPM/ADA/AGS/APhA/ASPC/NLA/PCNA Guideline on the Management of Blood Cholesterol: A Report of the American College of Cardiology/American Heart Association Task Force on Clinical Practice Guidelines. *Circulation*. 2019;139(25):e1082-e1143. doi:10.1161/CIR.0000000000000625

30. Powers WJ, Rabinstein AA, Ackerson T, et al. 2018 Guidelines for the Early Management of Patients With Acute Ischemic Stroke: A Guideline for Healthcare Professionals From the American Heart Association/American Stroke Association. *Stroke*. 2018;49(3):e46-e99. doi:10.1161/STR.0000000000000158

31. Konstantinides SV, Meyer G. The 2019 ESC Guidelines on the Diagnosis and Management of Acute Pulmonary Embolism. *Eur Heart J*. 2019;40(42):3453-3455. doi:10.1093/eurheartj/ehz726

32. Brignole M, Moya A, de Lange FJ, et al. 2018 ESC Guidelines for the diagnosis and management of syncope. *Eur Heart J*. 2018;39(21):1883-1948. doi:10.1093/eurheartj/ehy037

33. Regitz-Zagrosek V, Roos-Hesselink JW, Bauersachs J, et al. 2018 ESC Guidelines for the management of cardiovascular diseases during pregnancy. *Eur Heart J*. 2018;39(34):3165-3241. doi:10.1093/eurheartj/ehy340

34. Neumann FJ, Sousa-Uva M, Ahlsson A, et al. 2018 ESC/EACTS Guidelines on myocardial revascularization. *Eur Heart J*. 2019;40(2):87-165. doi:10.1093/eurheartj/ehy394

35. Williams B, Mancia G, Spiering W, et al. 2018 ESC/ESH Guidelines for the management of arterial hypertension. *Eur Heart J*. 2018;39(33):3021-3104. doi:10.1093/eurheartj/ehy339

36. Knuuti J, Wijns W, Saraste A, et al. 2019 ESC Guidelines for the diagnosis and management of chronic coronary syndromes: The Task Force for the diagnosis and management of chronic coronary syndromes of the European Society of Cardiology (ESC). *Eur Heart J*. 2020;41(3):407-477. doi:10.1093/eurheartj/ehz425

37. Brugada J, Katritsis DG, Arbelo E, et al. 2019 ESC Guidelines for the management of patients with supraventricular tachycardiaThe Task Force for the management of patients with supraventricular tachycardia of the European Society of Cardiology (ESC): Developed in collaboration with the Association for European Paediatric and Congenital Cardiology (AEPC). *Eur Heart J*. 2020;41(5):655-720. doi:10.1093/eurheartj/ehz467

38. Cosentino F, Grant PJ, Aboyans V, et al. 2019 ESC Guidelines on diabetes, pre-diabetes, and cardiovascular diseases developed in collaboration with the EASD: The Task Force for diabetes, pre-diabetes, and cardiovascular diseases of the European Society of Cardiology (ESC) and the European Association for the Study of Diabetes (EASD). *Eur Heart J*. 2020;41(2):255-323. doi:10.1093/eurheartj/ehz486

39. Mach F, Baigent C, Catapano AL, et al. 2019 ESC/EAS Guidelines for the management of dyslipidaemias: lipid modification to reduce cardiovascular risk: The Task Force for the management of dyslipidaemias of the European Society of Cardiology (ESC) and European Atherosclerosis Society (EAS). *Eur Heart J*. 2020;41(1):111-188. doi:10.1093/eurheartj/ehz455
