# Supplementary figures and images for: Intellectual conflicts of interest among cardiology and pulmonology clinical practice guidelines
Source: PLoS One. 2023 Jul 10;18(7):e0288349. doi: 10.1371/journal.pone.0288349 (PMC10332620; doi:10.1371/journal.pone.0288349)

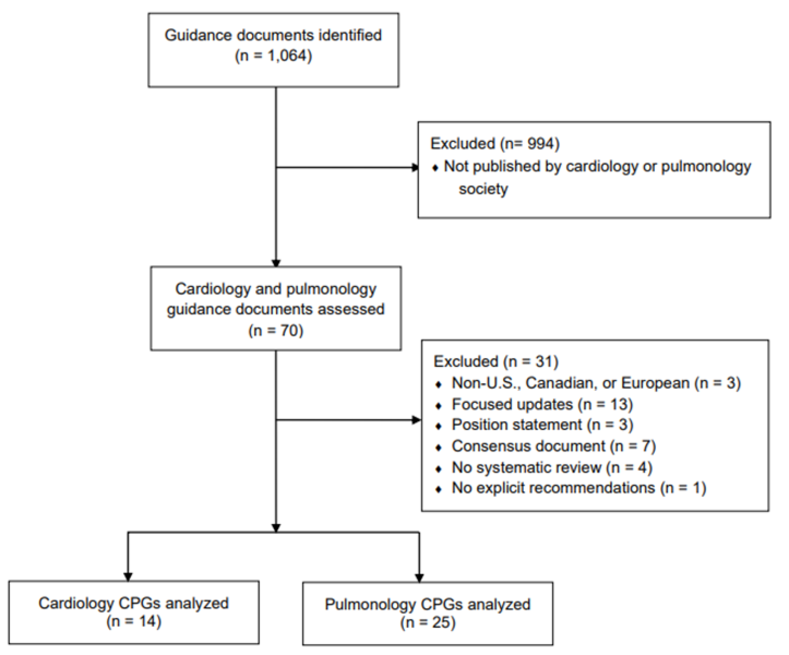

Supplement: S1 Fig — (TIF) [file pone.0288349.s002.tif]
