# Supplementary material for: Intellectual conflicts of interest among cardiology and pulmonology clinical practice guidelines
Source: PLoS One. 2023 Jul 10;18(7):e0288349. doi: 10.1371/journal.pone.0288349 (PMC10332620; doi:10.1371/journal.pone.0288349)
Supplement: Attachment — Submitted filename: iCOI_Response letter_PLOS One.docx [file pone.0288349.s003.docx]

March 22, 2022

Gladys Honein-AbouHaidar, PhD

Academic Editor

*PLOS ONE*

Dear Dr. Honein-AbouHaidar

Thank you for the opportunity to revise our manuscript “Intellectual conflicts of interest among cardiology and pulmonology clinical practice guidelines.” We appreciate the reviewers’ insightful comments and have responded to each point in detail below.

Sincerely,

J. Henry Brems, MD, MBE

Division of Pulmonary and Critical Care Medicine

Johns Hopkins University

**Reviewer 1**

| Reviewer Comment | Response |
| --- | --- |
| **Although the authors mention studies by Bero and Grundy (their references 32 and 33) in relation to the ability to quantify intellectual COI, they do not deal with the core of Bero’s and Grundy’s arguments that nonfinancial influences/interests may not fit a definition of conflicts of interest. Further, Bero and Grundy propose other methods, such as reflexivity as a tool that can be used that makes transparent and accounts for researchers' professional and personal identities. The authors need to deal in more detail with whether there is a distinction between COI and interests/influences beyond just saying that both financial and intellectual COI can be quantified.** | We thank the reviewer for highlighting this. A key criticism by Bero and Grundy is that a term such as “non-financial COI” is too broad and variably defined to be useful. Indeed, we agree with that point and believe our approach to intellectual COl is responsive to their argument. As suggested by Bero and Grundy, we specifically define COI by what it is (i.e. *intellectual* COI) rather than what it is not (i.e. *nonfinancial* COI). Moreover, rather than using “intellectual COI” as a vague analogy for “intellectual beliefs” we have restricted our definition of intellectual COI to three specific criteria, all of which could reasonably represent a secondary interest that a CPG panelist would have (i.e. to promote or uphold the validity of their own work)  We agree with the reviewer that these are important clarifications. As such, we have edited this section in the discussion as follows:  " Our results also address previous concerns surrounding inclusion of intellectual COI in CPG management. Some have previously argued that “non-financial COI” is too broad and is used to represent intellectual beliefs rather than to define actual conflicts of interests. (32–34) While these concerns remain valid for the term “non-financial COI,” our results demonstrate that the narrower “intellectual COI” can be well-specified with only a few criteria and is highly prevalent. Importantly, intellectual COI, as defined in our study, plausibly present a true COI rather than just intellectual beliefs because CPG panelists may have an interest in promoting their prior published work via CPG recommendation given the potential career and reputational benefits.” |
| **The authors declare that they don’t have any conflicts of interest to disclose but does that include intellectual COIs? For example, three of the authors (Helms, Davis and Clayton) published an article in PLoS One last year on COI policies among organizations producing CPGs. Does that constitute an intellectual COI?** | We appreciate the author bringing this up and admit it is a point we considered. While disclosure of COI is critical, there is also a need to balance what is disclosed (to Bero and Grundy’s point above) in order to not dilute and make COI disclosures meaningless.  In contrast to a CPG, we are not reviewing our prior work to produce a recommendation related to it. Thus, we are not sure there is a clear secondary interest that would produce a conflict here and did not disclose this as intellectual COI.  Nonetheless, we are more than happy to include a disclosure that we have previously published a paper about COI policies if the reviewer or editor would prefer. |
| **Why did the authors choose the January 1, 2018 to December 31, 2019 time frame?** | With the lack of other comprehensive CPG databases, we generated a 2-year sample of CPGs in order to aid comparability to prior systematic evaluation of CPGs that had used a similar time-frame (Norris et al, PLOS One 2011 Oct). Our collection of CPGs was used for a prior study as well (Brems et al, PLOS One, 2021 Apr). The specific window was chosen as project design and data collection began in 2020 for this study. We have added to the Methods to note this:  “Further, due to the lack of an existing comprehensive database, a two-year time frame was selected for this study for comparability to prior systematic evaluation of CPGs.(18,22)” |
| **Since the authors were potentially including Canadian CPGs, why didn't they search the database of CPGs maintained by the Canadian Medical Association?** | We appreciate the author bringing up this resource. We were not aware of this database from our review of prior studies on COI within CPGs, and while other databases may exist, we believe our search of three large, international databases was reasonably comprehensive—particularly in the absence of the National Guidelines Clearinghouse Database, which had been a previous standard in the literature.  However, given that no Canadian organization were included in our analysis, this is certainly a notable limitation of study. We have added the following to our discussion.  “It should be noted that, although we considered CPGs published by Canadian professional societies eligible for inclusion our study, we did not identify any that met full inclusion criteria. There may be important differences between Canadian, American, and European professional societies with respect to intellectual COI. Further studies are needed to confidently draw any conclusions about intellectual COI in Canadian CPGs.” |
| **How was authorship defined? For example, if there were CPG committee members named within the guideline but not explicitly listed as authors were they considered as authors? What about internal reviewers of the guidelines, were they considered as authors?** | We thank the reviewer for noting this. We only considered those who were explicitly labeled as authors. We excluded any other mentions of reviewers or panel members because these were non-standardized and their contribution were not always clear. Further, we reason that authors are the likely to be most involved in CPG production and thus the ones for whom COI matters most.  We have amended the Methods section to note:  “We assessed intellectual COI among all CPG authors and only considered those explicitly listed as authors by the CPG. We did not evaluate others listed only as reviewers or panelists because these were non-standardized and their contribution to CPG development was not always clear.” |
| **On page 12, the authors mention the percent of authors with one of 4 intellectual COI subtypes but earlier they only give three subtypes: i) authorship on a study reviewed by the CPG; ii) publication of a prior editorial; iii) membership on a prior CPG panel.** | We appreciate the reviewer catching this. We meant one of 3 subtypes and have corrected this error. |
| **Did the authors investigate whether CPG authors also had financial conflicts of interest? If both intellectual and financial COIs existed which one would have more of an effect or is that something that the CPG readers would have to determine?** | We agree this is an excellent question, but it was beyond the scope of our present study, in which our goal was to estimate the current prevalence of iCOI and associated management strategies in absence of prior data. It is an important consideration for both CPG developers and future research to understand the relationship between iCOI and financial COI, the frequency with which they co-occur, and which one may be more impactful.  We have edited the relevant section in the Discussion to state:  “Second, we did not investigate financial COI, so we cannot directly assess the prevalence or importance of financial COI relative to intellectual COI. However, even for financial COI, the thresholds used to define ‘significant’ or ‘allowable’ COI are arbitrary.(36) Given how common intellectual COI was in our study, CPG developers ought to consider and implement thresholds for it as well, even if further study is needed to refine those thresholds.” |
| **On page 16 (4th line) the authors say that one paper suggested that 15% of the recommendations of a CPG could have been biased by intellectual COI but Akl et al do not provide any information about how that figure was derived.** | This is a very important qualification. The estimate of 15% of recommendations being biased is not clearly data-driven, but in the absence of any prior estimates to our knowledge in the literature, it is a starting place to consider the concrete impact.  We have amended language within this section of the discussion to communicate the uncertainty of this estimate.  “Although there is a lack of evidence, it has been suggested before that CPG panels including a member with intellectual COI could have bias in 15% of their recommendations.(30) While that estimate could be incorrect, even 5% of recommendations’ being inappropriately biased could result in significant harm to patients given the high number of CPGs combined with the prevalence of intellectual COI.” |

**Reviewer 2 / Academic Editor**

| Reviewer Comment | Response |
| --- | --- |
| **The study covered CPGs published up to December 2019. Since then, different organizations emphasized the importance of addressing non-financial conflict of interest in CPGs to cite a couple: Alhazzani W, Lewis K, Jaeschke R, Rochwerg B, Møller MH, Evans L, Wilson KC, Patel S, Coopersmith CM, Cecconi M, Guyatt G, Akl EA. Conflicts of interest disclosure forms and management in critical care clinical practice guidelines. Intensive Care Med. 2018 Oct;44(10):1691-1698. & Nejstgaard CH, Bero L, Hróbjartsson A, Jørgensen AW, Jørgensen KJ, Le M, Lundh A. Conflicts of interest in clinical guidelines, advisory committee reports, opinion pieces, and narrative reviews: associations with recommendations. Cochrane Database Syst Rev. 2020 Dec 8;12(12. One would expect that disclosing intellectual COI would better addressed by methodologists and panelists of CPGs. It will be of an added value to examine whether recent CPGs up to 2022 had better management of COIs.** | We thank the reviewer for bringing up this point. We agree that the field may be evolving and that a comparison to the most recent guidelines would be of interest. However, we feel that such a comparison is beyond the scope of our study, in which our intention is to provide the first systematic evaluation of intellectual COI among more than 2 CPGs. Nonetheless, we agree some changes are likely, and evaluations of changes in prevalence and importance of intellectual COI over time are critical areas of future study.  This must be considered in the interpretation of our results and as a guide for future research. As such, we have included points on this in our discussion.  “Further, while we evaluated CPGs published in 2018 and 2019, some recent publications indicate that CPG developers may be paying increased attention to the intellectual COI.(36,37) The ACC—a major developer of cardiology CPGs—initiated a review of its CPG policies and procedures in 2020.(38) While intellectual COI was not mentioned explicitly in that report,(38) it is possible that the COI policy or composition of CPG panels could be shifting for the ACC and for CPG developers more broadly. Thus, future studies to evaluate shifts in prevalence and management of intellectual COI over time will be critical.” |
| **The authors indicated USA, Canada and Europe will be included in the CPG search. From the findings, none of the CPGs was Canadian. Were there no CPGs during that period or they were not included in the search?** | In our initial search, we did find 7 candidate guidance documents that were primarily produced by Canadian professional societies (6 by the Canadian Cardiovascular Society, 1 by the Canadian Cardiovascular Harmonized National Guideline Endeavour). However, none of these met our full inclusion criteria as they were all either position statements, focused updates, and/or lacked systematic reviews.  As based on our criteria, there were no Canadian organization identified.  We did add the following to the limitation section of the discussion in light of this:  “It should be noted that, although we considered CPGs published by Canadian professional societies eligible for inclusion our study, we did not identify any that met full inclusion criteria. However, there may be important differences between Canadian, American, and European professional societies with respect to COI management. Further studies are needed to confidently draw any conclusions about intellectual COI in Canadian CPGs.” |
