# Supplementary material for: Intellectual conflicts of interest among cardiology and pulmonology clinical practice guidelines
Source: PLoS One. 2023 Jul 10;18(7):e0288349. doi: 10.1371/journal.pone.0288349 (PMC10332620; doi:10.1371/journal.pone.0288349)
Supplement: Attachment — Submitted filename: iCOI_Response letter_PLOS One_6.12.docx [file pone.0288349.s004.docx]

June 13, 2023

Gladys Honein-AbouHaidar, PhD

Academic Editor

*PLOS ONE*

Dear Dr. Honein-AbouHaidar

Thank you for the opportunity to again revise our manuscript “Intellectual conflicts of interest among cardiology and pulmonology clinical practice guidelines.” We appreciate the comments from the reviewer and feel we have strengthened the manuscript by incorporating their feedback.

In addition, we have disclosed our past publication to our COI statement. Further, I have added an additional intellectual conflict of interest as I was invited last month (May 2023) to join the Ethics and Conflict of Interest Committee for the American Thoracic Society, which was one of the CPG-producing organizations included in this study. The study design, data collection, analysis, initial submission, and initial revision of this manuscript were all completed prior to my joining of that committee.

Sincerely,

J. Henry Brems, MD, MBE

Division of Pulmonary and Critical Care Medicine

Johns Hopkins University

**Reviewer 1**

| Reviewer Comment | Response |
| --- | --- |
| **The authors appear to be equating financial COI and intellectual COI creating a false equivalence. While both types of COI can lead to biases in CPGs, biases due to financial COI are all in the same direction, i.e., in favour of the product(s) being considered, whereas the same is not true of intellectual COI. This type of COI can bias decision either in favour or against the product(s) being considered.  Despite equating the two types of COI, the authors may be treating intellectual COI differently from financial COI. When financial COI is investigated it only typically covers the previous three years on the assumption (possibly incorrect) that interactions longer ago than 3 years will no longer bias people. In this study, in the case of intellectual COI, the authors have not put any time limit on when individuals put forward a point of view that could constitute an intellectual COI. Therefore, we do not know whether the COI occurred last year or 10 years ago.** | These are excellent points, and we absolutely agree that financial and intellectual COI have critical differences that must be considered in interpreting our study. Our manuscript would benefit from a clearer discussion of these nuances. As such, we have added the following sections to the Discussion:  “Important differences between intellectual COI and financial COI should be considered in interpreting our results. First, financial COI likely only biases individuals in favor of the intervention for which they have a conflict. However, intellectual COI may bias an individual either for or against an intervention depending on their specific conflict (e.g. whether their prior study found evidence supporting or refuting an intervention). Second, in practice, financial COI disclosures are often only requested from the past few years, although the exact time frame varies by organization.[31] We did not impose a time limit for intellectual COI in this study because it is unclear if or when intellectual COI truly become irrelevant. Particularly because CPGs may consider evidence from more than a decade prior, intellectual COI have potential to endure. Despite these differences between financial and intellectual COI, both represent a threat to the validity of CPGs, are recognized by the IOM, and merit explicit consideration from CPG developers.[2]” |
| **The authors need to acknowledge that the absence of one of the three types of intellectual COI does not mean that other CPG authors did not have specific points of view about the issues being considered in the CPGs. Financial COIs are much easier to discover than intellectual COIs, since the latter will only be revealed if the individual has published or presented a particular point of view.** | This is an important point and one we agree should be emphasized. These criteria cannot evaluate all intellectual biases nor all intellectual COI. However, they are concrete and could be easily used by CPG developers to better manage intellectual COI in a systematic fashion. We have added the following to the Discussion:  “The criteria used in our study cannot identify all potential intellectual COI or pre-existing viewpoints. However, they present a simple, concrete method that CPG-developers could use to better evaluate and manage intellectual COI.” |
| **The authors should explicitly acknowledge that they did not search the Canadian Medical Association’s database of 1700 CPGs. They offer two explanations for this absence. First, they say that they were not aware of the database from their review of prior studies on COI within CGPs. However, the CMA database is mentioned in at least two relatively recent peer reviewed studies (Elder et al. CMAJ 2020;192:e617-e625; Shnier et al. BMC Health Services Research 2016;16:383). Second, claiming that any relevant Canadian CPGs would have been included in the three databases that they did search is speculation.** | We appreciate this point and agree that it is an important limitation of our study that should be emphasized to readers. We have amended the following statement to the relevant section of the Discussion to state:  “Given the lack of an existing single, universal CPG database, this could reflect a limit of our sampling strategy, which did not include a database such as the Canadian Medical Association’s Infobase that may be more enriched for Canadian CPGs” |
